# Surgeons of Baltimore

**Published:** 1880-12

**Authors:** B. Bernard Browne

**Affiliations:** Baltimore


VOL. I.——*December 1880.*——NO. 12.

ARTICLE I.

[*Continued from November Number.*]

### SURGEONS OF BALTIMORE.

BY B. BERNARD BROWNE, M. D., OF BALTIMORE.

Prof. Richard Wilmot Hall (1823) tied the *carotid artery* for the cure of a fungus growth in the antrum, and although no operation was performed on the tumor itself, the disease was removed and the patient cured. Dr. Hall has been credited by very high authority (Dr. Gross) with ligating in 1830 the *anteria innominata*, but he did not really ligate it, for instead of passing the ligature around the artery, he merely transfixed it as will be seen by a careful perusal of the *post mortem*.

During the period from 1810, to 1840, great attention was attracted, to the surgery of the *Bloodvessels;* this was in great measure owing to the unusual amount of anatomical investigation and study in this direction, led off by Sir John and William Hunter, and followed up by Sir Charles Bell, Allan Burns and Sir Astley Cooper in Europe, and in this country by the celebrated and enthusiastic teacher, John D. Godman.

Prof. Nathan Ryno Smith, who had assisted in laying the foundation of the Jefferson Medical School at Philadelphia, was appointed in 1827 to the chair of surgery in the University of Maryland. In 1831 he edited the *Medical and Surgical Memoirs* of his father, Prof. Nathan Smith, in this work he published the first account of his own Lithotomy Instrument, which has become so well known since that time. He published in 1832 his work on the "*Surgical Anatomy*" of the arteries, which was received with much favor both at home and abroa d

In 1834 he reported 23 successive and successful cases of Lithotomy done with his own Lithotome.

It was also about this time, (1835,) that he devised his *suspensory apparatus* for the treatment of fractures of the lower extremity.

In 1860 he devised the *anterior suspensory apparatus* which is well known in this country and in Europe, it was used by both armies during the late war in this country. Prof. Harvey L. Byrd has devised a *wire gauze supporter* to be used in connection with this splint.

Dr. Smith is said to have operated 250 times for *lithotomy*.

The first complete *exterpation of the superior maxilla* done in Baltimore was by Prof. Christopher Johnston, in 1873, for Sarcoma. He has also operated successfully for *extrophy of the bladder* (1876) being the first case reported in Maryland. He and Prof. J. S. Michael have both operated successfully for close amputation of the penis for epithelioma.

Dr. Allan P. Smith has reported 52 successive cases of lithotomy without a death. One of the patients presented the rare deformity of a double penis and two distinct bladders.

Prof. J. J. Chisolm has successfully removed from the bladder, by lithotomy, a minnie ball which perforated the abdominal walls and lodged in the bladder.

The most difficult and heroic operation recorded in the annals ot surgery was performed February 25, 1878, by Prof. L. McLane Tiffany. "The first unsuccessful operation for the removal of a naso-pharyngeal polypus by temporary depression of both upper jaws," the operation was preceded by tracheotomy; chloroform was inhaled through the tracheotomy tube, the pedical of the tumor (which was pear shaped and four inches long) was half inch in diameter. He was the first surgeon south of Philadelphia to perform colotomy; he recorded 5 cases of "*Syphilis of the lung*" which were the first cases reported in America. He has excised a tumor of the Sciatic nerve, together with 5½ inches of that nerve. He has also contributed important papers on the "*cure of aneurism by flexion*," "*Deformity of the hip in the third stage of Morbus Coxraius*," "*Urethral Rheumatism*," and "*Surgical Kidney*."

Prof. O. J. Coskery has devised an extension apparatus for fractures of the leg which has been used with great success. He has also made an important modification in the use of the *Plaster of Paris* splint. He has recently extirpated from the neck of a large fibro-enchondroma, weighing 3 lbs. 10 oz. He and Dr. John R. Uhler have performed successfully simultaneous amputation of both lower extremities. Prof. Harvey L. Byrd has devised the "Combination operation" in amputation of the extremities.

In 1863 Dr. John R. Uhler published a paper on the Chemical Detection of lead and iron bullets in gunshot wounds. The paper has attracted considerable attention abroad and very favorable reports have been made of the results attained by the use of the *Chemical test.* Dr Lawrence F. Knight has devised an apparatus for treating transverse fractures of the patella.

An important contribution has been made to Gynecological Surgery by Dr. H. P. C. Wilson, who has devised the Antithermic Shield to be used in connection with Paquelins Thermo Cautery.

Dr wilson has performed successfully ovariotomy on a woman in the fourth month of pregnancy; she went to full term, and was delivered of a healthy child. He has also performed laparotomy for the removal of an extra uterine twin. The child lived.

Prof. Wm. T. Howard has also had occasion to perform laparotomy three times for the removal of the child in cases of ruptured uterus, in which he was called in consultation, he has written an exhaustive paper on the subject, which will shortly be published.

Dr. James H. Butler has performed laparotomy on a woman with deformed pelvis and ruptured uterus. The child died, but the woman recovered. He also performed cæsarean section on a woman who had a deformed pelvis and anchylosis of the hip. The coccyx has been removed for the cure of coccyodynia by Drs. H. P. C. Wilson, Wm. T. Howard, A. L. Knight and B. B. Browne.

*Ovariotomy* was first performed in Baltimore by Dr. John Murphy in 1849, and his patient recovered. Until the past few years there were few operations done here on account of the strong current of professional opinion against the justifiability of the operation; but of late years the statistics of results in Baltimore compare favorably with the northern cities. Battey's operation was done for the first time in Baltimore during the past year by Dr. Frank West.

Prof Howard has devised an improved bivalve speculum, and Prof. Erich a self-retaining one.

Dr. P. T. Knight has devised the obstetrical forceps that bear his name; they have been used with success in cases of difficult labor, with contracted pelvis, after failure with other forceps, and have been the means of delivering many living children who otherwise might have been the victims of craniotomy.

Prof. Harvey L. Byrd is the author of a "*Speedy Method in Asphyxia*," which has met with favorable comment in the various medical journals and elsewhere.

Prof. J. J. Chisolm published in 1861 a "*Hand Book of Military Surgery*," for the use of surgeons in the Confederate army. It ran through several editions, and was favorably received both at home and abroad. Dr. Chisolm has usd chloroform more than 10,000 times, and Dr. H. P. C. Wilson over 4,000 times without an unpleasant result in any case. In the *Annales D'Oculistique*, 1874-78; there are nineteen references to important papers written by Dr. Chisolm, this is more than is credited to any other American oculist.

Dr. Samuel Theobald has made many valuable contributions to ophthalmic and aural surgery, among which are his *needle holder* and *improved strabismus hook;* he has devised a series of lacrymal probes for strictures of the nasal duct; these probes are much larger than those that had previously been in general use. His article on Tinnitus Aurium has attracted much attention in this country and abroad.

Important contributions to the literature of syphilis and dermatology have been made by Prof I. E. Atkinson.

Baltimore, which was the first city in the world to project a successful railroad upon a large scale, which was the first city to light its streets with gas, and which was the first to receive a message by electric telegraph, has also been foremost and led the way in many important surgical operations and achievements.

